# Neuregulin 1 Signaling Attenuates Tumor Necrosis Factor α–Induced Female Rat Luteal Cell Death

**DOI:** 10.1210/endocr/bqae129

**Published:** 2024-09-23

**Authors:** Saswati Banerjee, Babayewa Oguljahan, Winston E Thompson, Indrajit Chowdhury

**Affiliations:** Department of Physiology, Morehouse School of Medicine, Atlanta, GA 30310, USA; Center for Laboratory Animal Resources, Morehouse School of Medicine, Atlanta, GA 30310, USA; Department of Physiology, Morehouse School of Medicine, Atlanta, GA 30310, USA; Department of Obstetrics and Gynecology, Morehouse School of Medicine, Atlanta, GA 30310, USA; Department of Obstetrics and Gynecology, Morehouse School of Medicine, Atlanta, GA 30310, USA

**Keywords:** neuregulin, signaling, corpus luteum

## Abstract

The corpus luteum (CL) is a transient ovarian endocrine structure that maintains pregnancy in primates during the first trimester and in rodents during the entire pregnancy by producing steroid hormone progesterone (P4). CL lifespan, growth, and differentiation are tightly regulated by survival and cell death signals through luteotrophic and luteolytic factors, including the epidermal growth factor (EGF)-like factor family. Neuregulin 1 (NRG1), a member of the EGF family, mediates its effect through ErbB2/3 receptors. However, the functional role of NRG1 in luteal cells (LCs) is unknown. Thus, this study investigated the role of NRG1 and its molecular mechanism of action in rat LC. Our experimental results suggest a strong positive correlation between steroidogenic acute regulatory protein (StAR) and NRG1 expression in mid-CL and serum P4 and estrogen (E2) production. In contrast, there was a decrease in StAR and NRG1 expression and P4 and E2 production with an increase in tumor necrosis factor α (TNFα) expression in regressing CL. Further in vitro studies in LCs showed that the knockdown of endogenous Nrg1 promoted the expression of proinflammatory and proapoptotic factors and decreased prosurvival factor expression. Subsequently, treatment with exogenous TNFα under these experimental conditions profoundly elevated proinflammatory and proapoptotic factors. Further analysis demonstrated that the phosphorylation status of ErbB2/3, PI3K, Ak strain transforming or protein kinase B (Akt), and ErK1/2 was significantly inhibited under these experimental conditions, whereas the treatment of TNFα further inhibited the phosphorylation of ErbB2/3, PI3K, Akt, and ErK1/2. Collectively, these studies provide new insights into the NRG1-mediated immunomodulatory and prosurvival role in LCs, which may maintain the function of CL.

The corpus luteum (CL) is a transient ovarian endocrine gland formed from the ovulated follicle under the influence of mid-cycle luteinizing hormone (LH) surge. CL is the primary source of progesterone (P4) during the first trimester in primates, and the entire pregnancy period in rodents, thus timely luteolysis is necessary for normal delivery (1-3). CL is a heterogeneous structure and composed of a mixture of cells consisting of not only steroidogenic luteal cells (LCs) but also nonsteroidogenic cells, including vascular endothelial cells, fibroblasts, and immune cells such as macrophage, neutrophils, eosinophils, T lymphocytes, and monocytes (2-4). LCs originate from granulosa cells (GCs), mainly as large LCs and small LCs from the theca cells (2, 3). Interestingly, CL lifespan, growth, and differentiation are tightly regulated both by survival and cell death signals through luteotrophic factors, including hormones (LH; human chorionic gonadotropins; prolactin; estrogen, E2; and prostaglandin E2), epidermal growth factor (EGF)-like peptide that promotes survival and P4 production, and luteolytic factors, such as PGF2a, and locally produce cytokines and chemokines that regulate them (5-8). The various luteotropic and luteolytic factors are produced both by steroidogenic and nonsteroidogenic cells in the CL and act as autocrine and paracrine mediators, which coordinate complex intracellular and extracellular events in modulating CL fate through cell migration and activation, proliferation, survival, and apoptosis, ultimately governing CL homeostasis and differentiation locally (4, 8-11).

Cytokines and chemokines are diverse pleiotropic immunoregulatory signaling proteins with a short half-life that are produced in a gonadotropin-dependent manner locally in optimal levels and secreted as chemotactic gradients in ovarian follicles and the CL (12-22). These cytokines and chemokines, including interleukins (ILs) and pleiotropic cytokine tumor necrosis factor α (TNFα), act in an autocrine-paracrine fashion and regulate many hallmark processes in ovarian physiology such as cellular survival and death, local and systemic inflammation, and CL functional regression (luteolysis) (1-4, 9, 13, 15, 16, 23-27). Studies have also demonstrated a diverse physiological function of TNFα and its receptors (TNFR1 and TNFR2) in follicular development, ovulation, CL formation and luteolysis, steroidogenesis, angiogenesis, and maintenance of early pregnancy (4, 16, 24-26, 28-30). Besides TNFα, the proinflammatory cytokines, namely IL-1, IL-6, and IL-8, play profound roles in this process (4, 28, 31-34). LCs and endothelial cells are the main sources of TNFα in CL, and its expressions are LH and P4 dependent (35, 36). The luteolysis is characterized by the decline in P4 synthesis and secretion, which precedes LC death and structural luteolysis of the CL (2, 3, 27). During functional regression of the CL, there is an increased level of TNFα expression and activation of different members of the caspase family with decreased expression of the steroidogenic acute regulatory protein (StAR) protein and P4 synthesis and secretion (1-3, 37, 38). StAR facilitates the transport of cholesterol from the outer to the inner mitochondrial membrane and serves as the rate-limiting step in P4 synthesis (39). The StAR protein is highly expressed in the early and mid-luteal phase of CL, whereas the expression of StAR declines along with the decrease in P4 synthesis and secretion during the regression of CL (1-3). Therefore, StAR, TNFα, and caspases are used as functional markers of CL during luteal development and luteolysis (37, 38). Thus, any CL defects, or luteal phase deficiency, contribute to decreasing P4 production and the subsequent inability to support a developing fetus (2, 3, 27, 38, 40). Despite abundant pieces of evidence, little is known about the physiological regulations and interactions of immunomodulatory molecules and cytokines during CL development and maturation. This is mainly because of the complex cellular composition of CL and their interrelationships.

Previous studies have suggested that gonadotropin-dependent transactivation of the EGF-family members network is indispensable for follicular development, maturation, and ovulation (13, 41-48). Neuregulin-1 (NRG1), a member of the EGF-like factor family, is gonadotropin-dependent, differentially expressed in antral and ovulatory follicles, and regulates a wide range of functions, including cytokine and chemokine expression and secretion, and supports cell survival, oocyte maturation, and P4 synthesis (42, 43, 45, 46, 48). Other studies have demonstrated that GC-specific *Nrg1* knockout mice (gcNrg1KO) showed ovarian and endocrine phenotypes like older women with follicular development blocked in bilayer secondary follicles and heterogeneous cells accumulated in the ovarian stroma (49). Moreover, studies have demonstrated that NRG1 attenuates tissue damage in acute brain injuries, inactivates inflammatory pathways associated with tissue damage during ischemic episodes, and supports cardiac functions and the development of heart, nerve, and muscles (50-57). The intracellular biological effects of NRG1 are mediated by a set of bona fide tyrosine kinase receptors, known as the EGF family receptors (ErbB) namely ErbB1, ErbB2, ErbB3, and ErbB4 (58). Binding to NRG1 triggers the homodimerization of ErbB4 and heterodimerization of ErbB2/3, ErbB2/4, and ErbB3/4 (59-61). The receptor dimerization leads to autophosphorylation and transphosphorylation of intracellular tyrosine kinase domain with activation of phosphatidylinositide 3 kinase (PI3K)–protein kinase B (Akt), Ras/extracellular signal-regulated kinase 1/2 (ERK1/2), mitogen-activated protein kinase (MAPK) cascade to initiate intracellular signaling events and govern distinct cell-fate decisions (42-46, 62-65).

Despite these pieces of evidence, the physiological role of NRG1 in the CL and LCs still needs to be defined. In the present study, we hypothesize that NRG1 signaling modulates IL expression in LCs, ultimately supporting LC survival and differentiation. To address these issues, we first determined the expression pattern of NRG1 with StAR and TNFα in pregnant rat CL and correlated with the circulating plasma levels of E2 and P4. Subsequently, we determined whether the inhibition of endogenous NRG1 affected LC survival in the presence of exogenous TNFα treatment. In further studies, ErbB2 and ErbB3 receptors were colocalized in the CL and demonstrated that endogenous NRG1-dependent activation of ErbB2/3, PI3K, AKT, and ERK attenuate the TNFα-dependent LC death.

## Materials and Methods

### Animal Model and Sample Collections

All experiments were approved by the Institutional Animal Care and Use Committee under the guidelines of the National Institutes of Health (NIH) and the US Department of Agriculture. Sprague-Dawley (SD) female rats (nonpregnant, aged 22 days, pregnant rats on gestational days 8 and 18) were purchased from Charles River Laboratories. Animals were given food and water ad libitum. They were kept under normal, environmentally relevant conditions (12 hours light:12 hours darkness), maintained automatically with lighting changes occurring at 0600 and 1800 hours and at room temperature (21-23 °C).

Body weights (BWs) were measured on day 26 of nonpregnant and day 12 and 22 pregnant rats, followed by anesthetization with isoflurane inhalation, and a cardiac puncture was performed to collect blood samples into serum separator tubes (BD). The serum was separated by centrifugation (6000*g* for 20 minutes) and stored at −80 °C until assayed for hormone levels. After that, rats were euthanized, and the number of fetuses were counted for pregnant rats. Then, the ovaries were collected under aseptic conditions and cleared from the surrounding fats. The CL were rapidly dissected from one ovary from each pregnant rat, whereas nonpregnant rat ovaries were immediately stored at −80 °C or until used for RNA and protein isolation. The contralateral ovaries of these animals were fixed for 24 hours in 10% (weight-to-volume ratio) buffered formalin (catalog No. 9400-1, VWR), dehydrated in 70% (volume-to-volume ratio) ethanol (catalog No. 01-335-389, Fisher Scientific), embedded in paraffin, and stored at room temperature until immunohistochemical (IHC) processing was performed.

### Serum Hormones Analysis

Serum P4 (catalog No. progesterone–mouse & rat) and estrogen (E2; catalog No. estradiol–mouse & rat) concentrations were measured by the Ligand Assay and Analysis Core at the Center for Research in Reproduction, University of Virginia. The limits of detection of the assays were E2, 5.0 to 3200 pg/mL, and P4, 0.30 to 80 ng/mL. Samples below the assay threshold were assigned the threshold value. Detailed information on the hormone analyses is available at https://med.virginia.edu/research-in-reproduction/ligand-assay-analysis-core/.

### Immunohistochemical Analysis and Hematoxylin and Eosin Staining

To elucidate the expression pattern of NRG1, ErbB2, and ErbB3 in nonpregnant and pregnant rat ovaries, they were colocalized along with von Willebrand factor (vWF) and StAR (42, 43). Paraffin-embedded nonpregnant and pregnant rat ovaries were serially sectioned (n = 10; 7-μm thick) from each ovary per rat (n = 3 rats per group), deparaffinized in xylene washes, rehydrated, and quenched with 3% hydrogen peroxide in methanol; nonspecific sites were blocked with 20% (volume-to-volume ratio) nonimmune serum/phosphate-buffered saline, and then incubated with primary antibodies as described in Table 1. Finally, ovarian sections were incubated with Alexa Fluor 594–conjugated goat anti-rabbit immunoglobulin G (red) and Alexa Fluor 488–conjugated goat anti-rat immunoglobulin G (green) secondary antibody (2 μg/mL; Molecular Probes). Negative controls were performed by omitting the primary antibody or using an isotype-matched control antibody derived from the same species. Slides were mounted using ProLong Gold Antifade reagent with DAPI (4′,6-diamidino-2-phenylindole) (Thermo Fisher Scientific). Mounted slides were examined using an Olympus microscope with an Optronics Magnifier digital camera (Olympus).

**Table 1.** List of antibodies used for Western blot analysis and immunohistochemistry

Peptide/Protein target	Name of antibody	Source	Species raised (monoclonal or polyclonal)	Research resource identifier (RRID)	Dilution used for WB and IHC
Neuregulin-1 (Nrg1)	Antineuregulin-1 (Nrg1)	Thermo Fisher Scientific	Mouse (polyclonal)	AB_10986946 http://antibodyregistry.org/AB_10986946	1:500 (WB) 1:50 (IHC)
vWF	Anti-vWF	Thermo Fisher Scientific	Rabbit (polyclonal)	AB_10642840 http://antibodyregistry.org/AB_10642840	1:200 (IHC)
StAR	Anti-StAR	Thermo Fisher Scientific	Rabbit (polyclonal)	AB_2115832 http://antibodyregistry.org/AB_2115832	1:500 (WB) 1:20 (IHC)
Bcl-XL	Anti–Bcl-XL	Cell Signaling Technology	Rabbit (monoclonal)	AB_2228008 http://antibodyregistry.org/AB_2228008	1:500
Bcl2	Anti-Bcl2	Thermo Fisher Scientific	Rabbit (polyclonal)	AB_2544570 http://antibodyregistry.org/AB_2544570	1:500
BAK	Anti-BAK	Cell Signaling Technology	Rabbit (monoclonal)	AB_2716685 http://antibodyregistry.org/AB_2716685	1:500
IL-1β	Anti–IL-1β	Thermo Fisher Scientific	Rabbit (polyclonal)	AB_2804633 http://antibodyregistry.org/AB_2804633	1:500
IL-6	Anti–IL-6	Thermo Fisher Scientific	Rabbit (polyclonal)	AB_794649 http://antibodyregistry.org/AB_794649	1:500
IL-17a	Anti–IL-17a	Thermo Fisher Scientific	Rabbit (polyclonal)	AB_10859178 http://antibodyregistry.org/AB_10859178	1:500
IL-10	Anti–IL-10	Thermo Fisher Scientific	Rabbit (polyclonal)	Catalogue # BS-20373R	1:500
IL-12	Anti–IL-12	Thermo Fisher Scientific	Rabbit (polyclonal)	AB_10857349 http://antibodyregistry.org/AB_10857349	1:500
Phospho-ErbB2	Anti-Phospho-ErbB2	Thermo Fisher Scientific	Mouse (monoclonal)	AB_2816212 http://antibodyregistry.org/AB_2816212	1:500
Total ErbB2	Anti-ErbB2	Origene	Mouse (monoclonal)	AB_2629050 http://antibodyregistry.org/AB_2629050	1:500 (WB) 1:50 (IHC)
Phospho-ErbB3	Anti-Phospho-ErbB3	Thermo Fisher Scientific	Rabbit (monoclonal)	AB_10985249 http://antibodyregistry.org/AB_10985249	1:1000
Total ErbB3	Anti-ErbB3	Thermo Fisher Scientific	Mouse (monoclonal)	AB_10979685 http://antibodyregistry.org/AB_10979685	1:500 (WB) 1:100 (IHC)
Phospho-AKT	Anti-Phospho-AKT	Cell Signaling Technology	Rabbit (monoclonal)	AB_2315049 http://antibodyregistry.org/AB_2315049	1:500
Total AKT	Anti-Total AKT	Cell Signaling Technology	Rabbit (monoclonal)	AB_329827 http://antibodyregistry.org/AB_329827	1:500
Phospho PI3 kinase p85 alpha/gamma	Anti-Phospho PI3 kinase p85 α/γ	Thermo Fisher Scientific	Rabbit (polyclonal)	AB_10855760 http://antibodyregistry.org/AB_10855760	1:200
PI3 Kinase p85	Anti-PI3 Kinase p85	Cell Signaling Technology	Rabbit (polyclonal)	AB_659889 http://antibodyregistry.org/AB_659889	1:1000
p44/42 MAPK (Erk1/2)	Anti-Phospho-p44/42 MAPK (Erk1/2) (Thr202/Tyr204)	Cell Signaling Technology	Rabbit (monoclonal)	AB_2315112 http://antibodyregistry.org/AB_2315112	1:1000
Total 44/42 MAPK (Erk1/2)	Anti-Total 44/42 MAPK (Erk1/2)	Cell Signaling Technology	Rabbit (monoclonal)	AB_390779 http://antibodyregistry.org/AB_390779	1:1000
β-Actin	Anti–β-Actin	Cell Signaling	Rabbit (monoclonal)	AB_330288 http://antibodyregistry.org/AB_330288	1:1000

Abbreviations: ERK, extracellularly regulated kinase; IHC, immunohistochemistry; IL, interleukin; MAPK, mitogen-activated protein kinase; StAR, steroidogenic acute regulatory protein; vWF, von Willebrand factor; WB, Western blot.

For histological analysis, ovarian sections were deparaffinized and stained with hematoxylin for the nuclei and eosin for cytoplasm and extracellular matrix (Sigma–Aldrich) at room temperature (Histology Core, Georgia Institute of Technology). The number of preantral, antral, preovulatory follicles, and CL per ovary were counted in 3 representative sections at least 20 μm apart. Follicles and CL were counted in the ovarian sections with approximately similar sizes among all groups.

The representative photomicrographs were arranged using Adobe Photoshop (Adobe Systems) without any further adjustment to maintain the true nature of the findings and create the final figures.

### Luteal Cell Culture

Immortalized rat luteal cells conditionally (GG-CL, referred to as LC in the text) (Applied Biological Materials Inc [ABM Inc]) was purchased and routinely maintained in Prigrow II medium (TM002, ABM) supplemented with 10% fetal bovine serum (FBS, Life Technologies) and 1% Pen-Strep (a mixture of penicillin G and streptomycin) (Life Technologies) in ECM-coated (G422, ABM Inc) tissue culture plates maintained at 33 °C in a humidified atmosphere containing 5% CO_2_.

### Transient Transfection of Small Interfering Neuregulin 1 and Reverse Transcription–Polymerase Chain Reaction

Based on our previous studies (42), NRG1 (GenBank: DQ176766.1) expression was knocked down in rat LCs using transient transfection with small interfering NRG1 (siNRG1, sequence available on request) or scramble1 (AllStars Negative siRNA AF 488, catalog No. ID:1027292, Qiagen) and scramble2 (negative control siRNA, catalog No. ID: 1022076, Qiagen) at 70% to 80% confluence by using Lipofectamine RNAiMAX Reagent (Life Technologies) in serum-free media. To evaluate the efficiency, specificity, and toxicity effects of siNRG1 on LCs, 3 different concentrations (10, 25, and 50 nM) of siNRG1 along with scrambles (scramble1 and scramble2) siRNAs and a nontransfected control were used. The transfected cells were incubated at 33 °C for 6 hours; after that, the media was replaced with media containing 1% FBS. The validation of dose and time for siNRG1 was based on our previous studies (42). The degree of NRG1-knockdown was analyzed using reverse transcription–polymerase chain reaction (PCR) in the nontransfected, scramble1, scramble2, and siNRG1-transfected LCs post transfection performance at 24 hours. According to the manufacturer's instructions, total RNA was extracted using Qiazol Reagent (Qiagen). Reverse transcription was performed using an iScript [complementary DNA] cDNA synthesis kit (Bio-Rad Laboratories). Quantitative PCR was carried out using SsoAdvanced Universal SYBR Green Supermix (Bio-Rad Laboratories) on a CFX Connect Real-Time PCR Detection System (Bio-Rad Laboratories). The sequences of the primer set used for the analysis are as follows: Nrg1, 5′-TGAAGGACCTGTCAAACGCG-3′ (forward, F) and 5′-TGCTCCTACTCAGGCAGAGA-3′(reverse, R); and 18S ribosomal RNA (18s rRNA), 5′ GCA ATT ATT CCC CAT GAA CG 3′ (F) and 5′ GGC CTC ACT AAA CCA TCC AA 3′ (R). The relative expression of the gene was calculated using the 2-ΔΔCT method. 18S Ribosomal RNA was used as an internal standard for normalization for each sample to obtain delta-*CT* (Δ*CT*). The normalized *CT* was then calibrated to control cell samples to obtain delta-delta-*CT* (ΔΔ*CT*) and used to calculate the relative fold expression of the specific gene. Effective transfection resulted in NRG1 knockdown of approximately 70% or greater. Plates displaying less than 60% NRG1 knockdown efficiencies were not considered for further analysis. Moreover, the reduction in NRG1 transcript expression was analyzed by Western blot (WB) since messenger RNA (mRNA) levels do not always correlate with protein levels whose protein products have long half-lives. Moreover, the cell viability was analyzed under these experimental conditions.

### Treatment of Tumor Necrosis Factor α and Cell Survival Analysis

LCs were grown in 6-well plates and transfected with scramble (scramble1) or siNRG1 as described earlier, followed by serum-starved cells in media with 0.2% FBS for 24 hours. Then, cells were washed once with phosphate-buffered saline and treated with TNFα (10 and 50 ng/mL) (R&D Systems Inc) for 24 hours. Live-cell phase-contrast pictures were taken to assess the survival status of LCs at 24 hours posttreatment. The dose and time of treatment for TNFα are based on apoptosis assay. The percentage of apoptosis was determined by nuclear staining with Hoechst 33 248 stain (12.5 ng/mL, Sigma-Aldrich) as described previously by Chowdhury et al (2017) (43). At least 250 to 300 cells were counted for each time point. After that, cells were harvested for RNA and protein isolation, and analysis.

### Caspase-3/7 Assay

After TNFα treatments, the caspase-3 and -7 activities were measured in living cells using the CellEvent Caspase 3/7 reagent (catalog No. R37111, Thermo Fisher Scientific) according to the manufacturer's guidelines. In brief, the media was removed, and the CellEvent Caspase 3/7 reagent was added and incubated at 37 °C for 30 minutes. The nucleus was counterstained with DAPI at 37 °C for 15 minutes. After that, caspase 3/7 expression was observed under an Olympus microscope with an Optronics Magnifier digital camera (Olympus) using the filter used for fluorescein isothiocyanate (502-530 nm) and DAPI (372-456 nm).

### Western Blot Analysis

The cells were lysed in an ice-cold lysis buffer (mammalian protein extraction reagent, M-PER–containing protease inhibitor) (Thermo Fisher Scientific) and followed by incubation for 30 minutes on ice and centrifugation at 10 000*g* for 15 minutes at 4 °C. The supernatant was transferred to new tubes, and protein measurement was performed using Coomassie plus protein assay reagent (catalog No. 23238, Thermo Fisher Scientific) and bovine serum albumin standard (catalog No. 23208, Thermo Fisher Scientific) as the standard. Samples were denatured by adding 4× LDS sample buffer (Life Technologies), followed by boiling for 5 minutes. A total of 20 µg of protein was separated on 12% NuPAGE Novex Bolt Bis-Tris Plus Gels using NuPAGE MES SDS Running Buffer (Life Technologies) and transferred onto nitrocellulose membranes using iBlot 2 Transfer Stacks (Life Technologies). Blots were incubated for 1 hour at room temperature in 5% nonfat dry milk to block nonspecific binding. Blots were then incubated overnight at 4 °C with primary antibodies, as described in Table 1. After washing and probing with the appropriate secondary antibodies, protein-antibody complexes were visualized using SuperSignal West Pico detection reagent (Thermo Fisher Scientific). Quantification of the scanned images was performed using NIH Image version 1.61 software.

### Statistical Analysis

All experimental data are expressed as mean ± SEM of 3 independent experiments (n = 3), using a minimum of 3 animals per time point. In each experimental group, variation among groups was analyzed by analysis of variance or unpaired *t* test using GraphPad PRISM software (GraphPad Software Inc). Multiple comparisons were analyzed using the student-Newman-Keuls test. Differences were considered statistically significant at *P* less than or equal to .05. The correlation coefficient between NRG1, StAR, TNFα, E2, and P4 was determined by linear regression analysis.

## Results

### Neuregulin 1 Expression in Pregnant Rat Corpus Luteum and Luteal Cells

**Figure 1.**
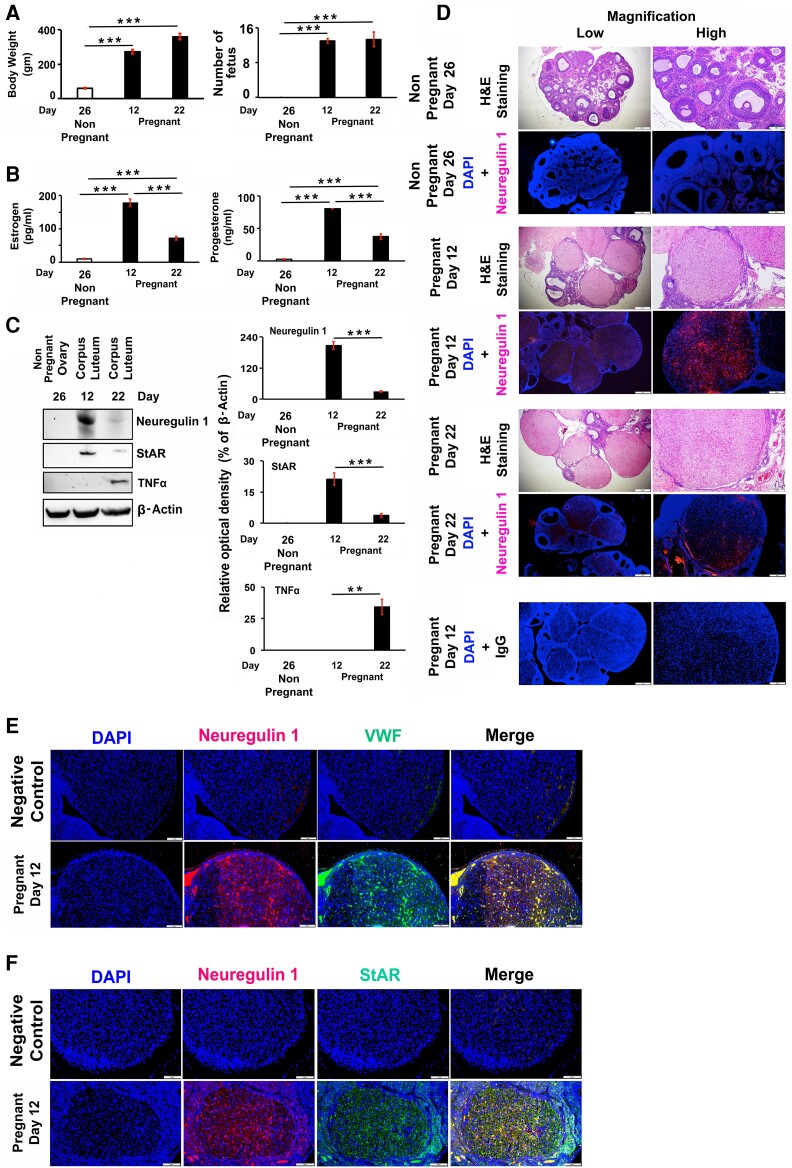
Neuregulin 1 (NRG1) expressions in rat corpus luteum (CL). A, Bar graphs show the changes in body weights of nonpregnant (day 26) and pregnant (day 12 and 22) rats and fetus numbers in pregnant rats. B, Bar diagrams represent the mean values of blood serum estrogen (E2) and progesterone (P4) in nonpregnant and pregnant rats. C, Representative Western blot (WB) analysis of protein levels of NRG1, steroidogenic acute regulatory protein (StAR), and tumor necrosis factor α (TNFα) expressions in the nonpregnant ovary and pregnant CL. Equal amounts of protein were used to each lane. β-Actin was used as an internal control. Bar diagrams represent the densitometric analyses of protein in WBs. D, Representative photomicrographs view of sections of nonpregnant, and pregnant rat ovaries stained with hematoxylin-eosin (H&E) and immunolocalization of endogenous NRG1 with Alexa Fluor 594–labeled (red) secondary antibody. The nucleus was stained with DAPI (4′,6-diamidino-2-phenylindole) (blue). E, Representative view of sections of pregnant rat ovaries on day 12 with immunolocalization of NRG1 and Von Willebrand factor (vWF) with Alexa Fluor 594– (red) and Alexa Fluor 488– (green) labeled secondary antibodies, respectively. The nucleus was counterstained with DAPI (blue). F. Representative view of sections of pregnant rat ovaries on day 12 with immunolocalization of NRG1 and StAR with Alexa Fluor 594–labeled (red) and Alexa Fluor 488–labeled (green) secondary antibodies, respectively. The nucleus was counterstained with DAPI (blue). All the data and numerical values (as mean ± SEM) are represented from 3 independent (n = 3) experiments performed for each group. Asterisks represent unpaired *t* test, **P less than or equal to .001; ***P less than or equal to .0001.

The pregnant rats' BW was taken, followed by pregnant rats being euthanized, and fetus numbers counted since intrauterine growth is most rapid during pregnancy due to an increase in the weight of the fetus and the mother. There was a significant increase in BW of pregnant rats on days 12 and 22 compared to nonpregnant rats, which correlates positively with the number of pregnant rat fetuses (Fig. 1A). The number of fetuses varies between 11 and 14 for each pregnant rat. The serum P4 and E2 concentrations in pregnant rats were significantly (*P* < .05) increased on day 12 and decreased on day 22 (Fig. 1B), suggesting that a matured CL had already formed and luteolysis started, respectively, as demonstrated previously (66). In contrast, a low, insignificant level of P4 and E2 in serum was detected in nonpregnant 26-day-old rats.

Next, we examined the expression pattern of NRG1, StAR, and TNFα in the nonpregnant ovary and CL, respectively. There was a statistically significant (*P* < .05) upregulation of NRG1 and StAR expressions and undetectable TNFα on day 12 CL, whereas there was a significantly (*P* < .05) lower expression of NRG1 and StAR and higher expression of TNFα on the day 22 CL (Fig. 1C). In contrast, there were no expressions of NRG1, StAR, and TNFα in nonpregnant ovaries. In addition, the expression of StAR in the pregnant rat CL was positively correlated with the serum levels of E2 (Y = 0.002 X –0.145; *R*^2^ = 0.987; *F*(1,4) = 297.35; *P* < .001), and P4 (Y = 0.0043 × X –0.154; *R*^2^ = 0.96; *F*(1,4) = 99.08; *P* < .001). Similar to StAR expressions, the expressions pattern of NRG1 in the pregnant rat CL were also positively correlated with the serum levels of E2 (Y = 0.018 X –1.177; *R*^2^ = 0.99; *F*(1,4) = 334.91; *P* < .001), and P4 (Y = 0.038 × X −1.273; *R*^2^ = 0.97; *F* (1,4) = 142.42; *P* < .001), and StAR (Y = 0.113 X –0.0112; *R*^2^ = 0.99; *F*(1,4) = 722.87; *P* < .001) expressions. In contrast, the expressions pattern of TNFα in the pregnant rat CL were negatively correlated with the serum levels of E2 (Y = −374.243 X –246.696; *R*^2^ = 0.65; *F*(1,4) = 7.52; *P* < .05), P4 (Y = −164.781 X –115.587; *R*^2^ = 0.57; *F*(1,4) = 5.25; *P* < .084), StAR (Y = −0.831 X –0.382; *R*^2^ = 0.75; *F*(1,4) = 12; *P* < .03), and NRG1 (Y = −7.167 X –3.419; *R*^2^ = 0.72; *F*(1,4) = 10.28; *P* < .03) expressions.

In further analysis, the ovarian sections of nonpregnant ovary stained with hematoxylin-eosin (H&E) revealed normal ovarian stroma with only a few numbers of preantral and antral follicles and no CL, whereas 12- and 22-day pregnant rats had preantral follicles and multiple CLs per ovary (Fig. 1D). However, the 22-day pregnant rat CL showed a patchy, often marked, mononuclear inflammatory cell infiltration, suggesting features of luteal regression. Moreover, IHC studies revealed a cell-specific spatial expression pattern of NRG1 in CL. On day 12, pregnant rats showed an intense NRG1 immunostaining throughout the CL, compared with a very low expression pattern of NRG1 on day 22 pregnant rat CL (see Fig. 1D). In further analysis, NRG1 was immunolocalized with von Willebrand factor (vWF), an endothelial marker, and StAR, a steroidogenic marker, to represent the LCs' specific expression of NRG1. Fig. 1E and 1F show that NRG1 is intensely colocalized with vWF in endothelial and LCs on day 12 pregnant rat CL, whereas StAR is intensely colocalized with NRG1 to LCs only. These IHC results showed that NRG1 is expressed throughout the CL, including LCs.

### Knockdown of Neuregulin-1 in Luteal Cells

**Figure 2.**
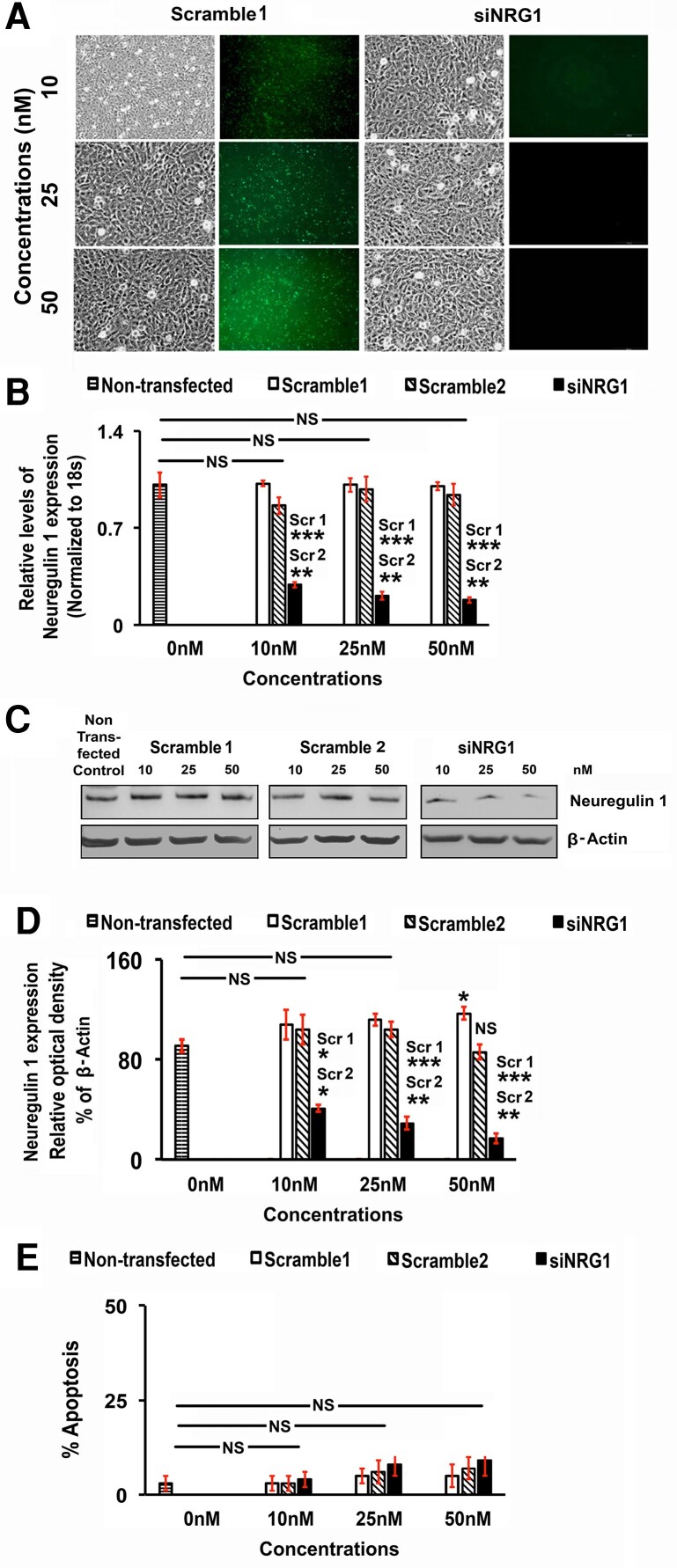
Knockdown of neuregulin 1 (Nrg1) in rat luteal cells (LCs). LCs were transiently transfected with different concentrations (10, 25, and 50 nM) of small interfering NRG1 (siNRG1) RNA or scramble1 (negative control with AF-488) and scramble2 (negative control) siRNA along with a nontransfected control at 70% to 80% confluency using RNAiMAX Reagent according to the manufacturer's instructions. After that, cells were maintained in serum-free media and harvested at 24 hours. A, Representative live-cell images (Bright field), and green-fluorescence (scramble1) were taken under an inverted microscope at 200× magnification at 24 hours post treatment to represent the morphological changes of cells. B, Bar graph demonstrates NRG1 messenger RNA (mRNA) expression levels detected by real-time quantitative polymerase chain reaction in the nontransfected control, scramble1, scramble2, and siNRG1 transfected LCs. The levels of mRNA were normalized to 18s ribosomal RNA (18s rRNA). C, Representative Western blot (WB) analysis of NRG1 protein expression levels in the nontransfected control, scramble1, scramble2, and siNRG1-transfected LCs at 24 hours post treatment. To each lane of the gel equal amounts of protein were applied. β-Actin was used as an internal control. D, Bar diagram represents the densitometric protein analyses in WB. E, The cells displaying the percentage of nuclear morphologic changes at 24 hours post treatment demonstrating apoptosis. All bar graphs represent the mean ± SEM of results from 3 individual experiments (n = 3). Asterisks represent unpaired t test, *P less than or equal to .01; **P less than or equal to .001; ***P less than or equal to .0001; NS, not significant. Scr1 or Scr2 with stars (**/***) represents the siNRG1 group comparison with respective scramble1 (Scr1) or scramble2 (scr2) group.

To directly test the potential physiological role of NRG1 on LCs, the expression of endogenous NRG1 was knocked down by transient transfection using siNRG1 in vitro. More than 70% of scramble-transfected LCs were homogenously transfected with uniform green immunofluorescence in culture without any adverse effects on cellular morphology (Fig. 2A). The phase-contrast photomicrograph showed both scramble- and siNRG1-transfected LCs have typical epithelial-like morphology with no apparent morphological differences post transfection. A dose-response study demonstrated that the selected siNRG1 dose significantly downregulated the expression of NRG1 at mRNA and protein levels in LCs compared to the scramble group (Fig. 2B-2D). The siNRG1 significantly and consistently knocked down NRG1 in mRNA levels (∼77%, 80%, and 82% in 10, 25, and 50 nM siNRG1 concentrations) at 24 hours in LCs. There were no significant differences in knockdown efficiencies of NRG1 at mRNA and protein levels between 25 and 50 nM siNRG1 concentrations. The scramble-transfected LCs had no adverse effects on NRG1 expression at the mRNA and protein levels when compared with the nontransfected control group and the scramble1 and scramble2-transfected groups under these experimental conditions (Fig. 2B-2D). However, siNRG1 knockdown efficiency was significantly higher compared to the scramble1 group both at the mRNA and protein levels. The functional effects of siNRG1 knockdown were further analyzed by evaluating cell survival since NRG1 is a prosurvival factor in different cell types, including GCs (42, 43). There was no significant apoptotic cell death in scramble1-, scramble2-, or siNRG1-transfected LCs post transfection at 24 hours compared to a nontransfected control group (Fig. 2E). Based on siNRG1 dose response on NRG1 expressions and prosurvival studies in LCs, we selected a 25-nM dose of siNRG1 along with scramble1 (represented as a scramble in the manuscript) group for the detailed studies.

### Knockdown of Endogenous Neuregulin 1 Promotes the Proapoptotic Effects of Exogenous Tumor Necrosis Factor α in Luteal Cells

We first determined the optimum concentration of TNFα for the induction of apoptosis in LCs. In brief, LCs were grown in media with serum to 95% to 100% confluence. After that, LCs were treated with different doses (1, 10, 50, and 100 ng/mL) of TNFα for 24 hours in serum-free media. Apoptotic analysis showed that TNFα potently and significantly caused apoptotic LC death in a dose-dependent manner (% apoptosis, 8 ± 0.58, degree of freedom [df] = 4; *P* < .004**; 13.67 ± 4, df = 4; *P* < .005**; and 52.67 ± 5.0, df = 4; *P* < .0001*** at 10, 50, and 100 ng/mL TNFα dose, respectively, compared to untreated control 3 ± 0.58 group) except the 1 ng/mL TNFα dose (% apoptosis, 3.33 ± 0.33, df = 4; *P* < .64, not significant). Based on these results, we selected 10-and 50-ng/mL TNFα doses for the detailed studies. The 100-ng/mL TNFα dose was excluded from the detailed studies due to greater than 50% apoptotic LC death at 24 hours.

**Figure 3.**
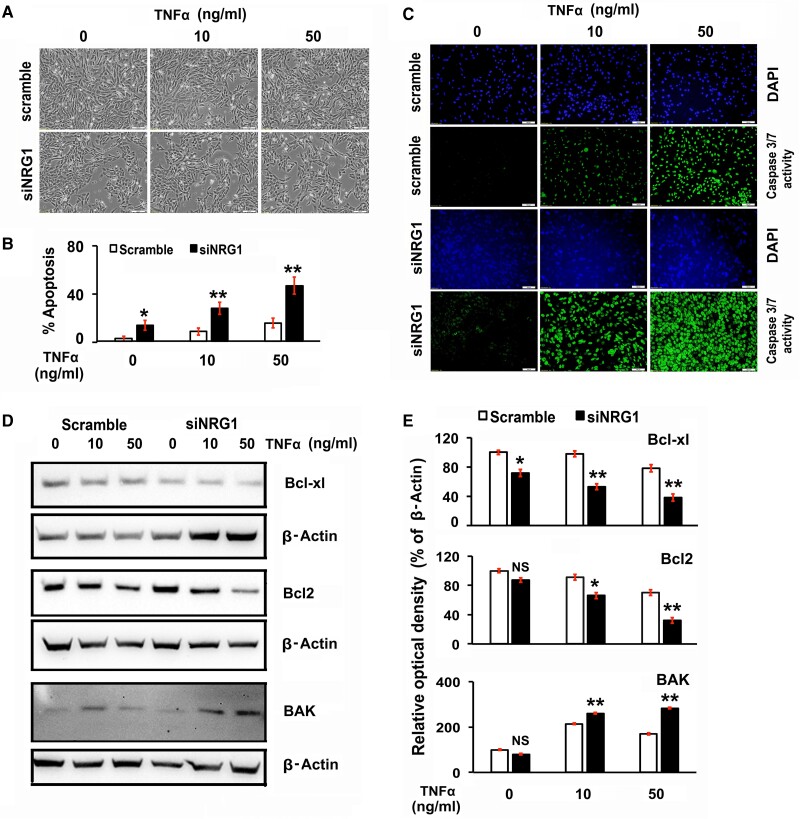
Effects of knockdown of endogenous neuregulin 1 (NRG1) on exogenous treatment of tumor necrosis factor α (TNFα) in rat luteal cells (LCs). LCs were grown at 70% to 80% confluency and transiently transfected with small interfering NRG1 (siNRG1) or scrambled RNA. Thereafter, cells were maintained in serum-starved media and treated with TNFα at different concentrations (10 and 50 ng/mL) for 24 hours. A, Representative pictures are showing the live-cell morphological changes at 24 hours post treatment taken under an inverted microscope at 200× magnification. B, The number of healthy and apoptotic cells quantified were expressed as a percentage of the total cells by counting without sample identity as blinded. A bar graph represents the percentage of cells displaying nuclear morphologic changes during apoptosis. C, Representative images of Caspase-3/7 activation at 24 hours post treatment with fluorescence imaging using Caspase-3/7 green detection reagent with FITC/Alexa Fluor 488. The nucleus was counterstained with DAPI (4′,6′-diamidino-2-phenylindole) (blue). D, Representative Western blot (WB) analysis of Bcl2, Bclxl, and Bak protein levels in LCs at 24 hours post treatment. To each lane of the gel equal amounts of protein were applied. β-Actin was used as an internal control. E, Bars diagrams represent the densitometric analyses of protein in WBs. All bar graphs represent the mean ± SEM of results from 3 individual experiments (n = 3). Asterisks represent unpaired *t* test, *P less than or equal to .01; **P less than or equal to .001; ***P less than or equal to .0001; and NS, not significant.

To investigate the effects of NRG1 on LCs, we treated serum-starved LC culture with or without endogenous NRG1 in the presence of exogenous cytokine TNFα (Fig. 3A and 3B). The endogenous NRG1 knockdown LCs treated with both doses of exogenous TNFα showed significantly higher numbers (siNRG1 group, ∼15%, 30%, and 50%; *P* < .05) of cells with apoptotic morphology, including exhibiting cell detachment, loss of cell processes, membrane shrinkage with the curling of cells, and formation of apoptotic bodies when compared with the parallel scrambled group treated with exogenous TNFα (∼4%, 10%, and 17% apoptosis; *P* < .05) (67) (see Fig. 3A and 3B).

TNFα indirectly promotes inflammation by inducing cell death (68). TNFα activates downstream signaling through 2 cognate receptors, TNF receptor 1 (TNFR1) and TNFR2, which have different intracellular signaling pathways (68). The TNF-TNFR1 complex activates the mitogen-activated protein kinase (MAPK) signaling cascade, which promotes and exacerbates inflammation by inducing cell death or apoptosis through the upregulation of proinflammatory genes transcription and activation of effector caspases (68). To determine the mechanism by which TNFα in LCs induces cell death, the levels of the effector caspases 3/7 activities were analyzed. The expression of caspase 3/7 activity was increased with TNFα treatment both in scrambled and siNRG1 LC groups (Fig. 3C). In contrast, TNFα treatment profoundly elevated the caspase 3/7 activity in Nrg1 knockdown LCs in a dose-dependent manner compared to the scrambled-treated group. At the higher dose (50 ng/mL for 24 hours) of TNFα treatment, NRG1-knockdown LCs showed higher expression of active caspase-3/7 when compared with the scrambled-treated group. The caspase-3/7 activity was corroborated by NRG1-knockdown LC morphological analysis in the presence or absence of TNFα treatment.

In further studies, to explore the mechanism by which endogenous NRG1 prevents apoptosis in LCs treated with TNFα, we examined the relative expression levels of proapoptotic and antiapoptotic proteins in the B-cell lymphoma (Bcl)-family. The Bcl protein family comprises a broad group of prosurvival factors, including Bcl2 and Bclxl, and proapoptotic factors, including Bak proteins (69, 70). Any intracellular imbalance of the proapoptotic vs prosurvival proteins ratio tilts the scales toward cell death through the activation of the downstream effector proteolytic cascade such as caspases, resulting in cell death (69, 70). Although NRG1 knockdown did not affect Bcl2 and Bak expression, there were significantly enhanced levels of Bak and decreased levels of Bcl2 and Bclxl protein expression in TNFα-treated LCs (Fig. 3D). Interestingly, when LCs were treated with TNFα in the presence of endogenous NRG1 (scrambled group), there were significantly higher Bcl2 and Bclxl levels and lower levels of Bak compared with that of the TNFα-treated NRG1 knockdown group. These results were corroborated by the quantitative expression analysis (Fig. 3E) and suggest the role of NRG1 as a prosurvival factor in LC.

### Knockdown of Endogenous Neuregulin-1 With the Treatment of Exogenous Tumor Necrosis Factor α in Luteal Cells Promotes Differential Expression of Interleukins and Steroidogenic Acute Regulatory Protein

**Figure 4.**
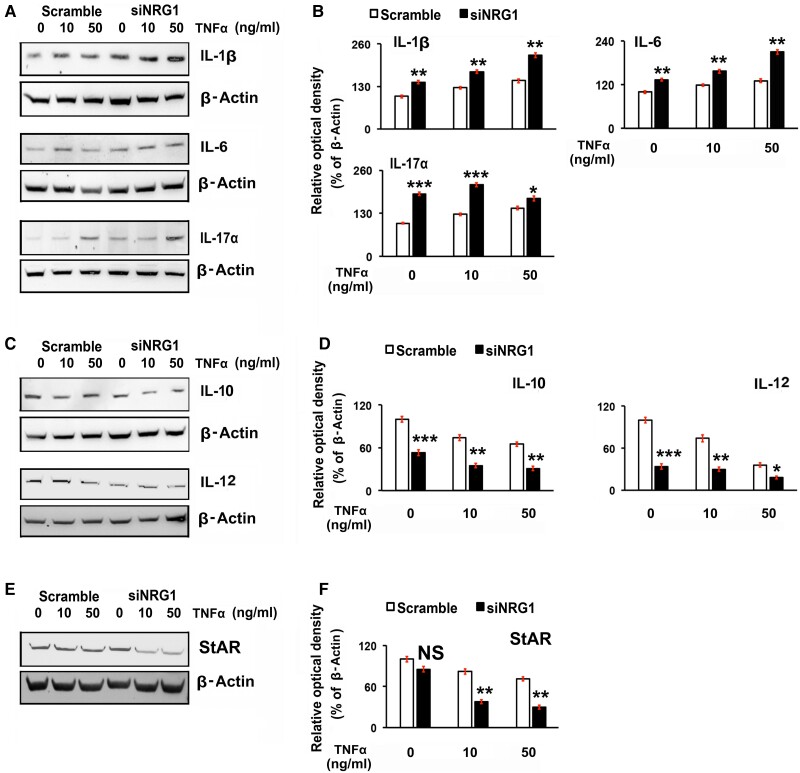
Effects of knockdown of endogenous neuregulin 1 (NRG1) on exogenous treatment of tumor necrosis factor α (TNFα)-dependent expressions of interleukins (ILs) and steroidogenic acute regulatory protein (StAR) in rat luteal cells (LCs). LCs were grown at 70% to 80% confluency followed by transiently transfected with small interfering-NRG1 (siNRG1) or scrambled RNA. After that, cells were maintained in serum-starved media and treated with TNFα at different concentrations (10 and 50 ng/mL) for 24 hours. Representative Western blot (WBs) analysis of A, IL-1β; IL-6; and IL-17a; C, IL-10 and IL-12; and E, StAR protein levels in LCs at 24 hours post treatment. To each lane of the gel equal amounts of protein were applied. β-Actin was used as an internal control. Bar diagrams B, D, and F represent the densitometric protein analyses in WBs. All bar graphs represent the mean ± SEM of results from 3 individual experiments (n = 3). Asterisks represent unpaired *t* test, *P less than or equal to .01; **P less than or equal to .001; ***P less than or equal to .0001; and NS, not significant.

To further understand the role of endogenous NRG1 on proinflammatory and anti-inflammatory ILs expression in LCs, selective IL (IL-1β, IL-6, IL-10, IL-12, and IL-17α) expression was analyzed. The ILs are an integral part of the signaling network that are produced differentially during physiological and pathophysiological processes (71). IL1β, IL-6, and IL-17α are proinflammatory cytokines of the innate immune system produced during tissue damage or in response to inflammatory stimuli (71, 72), whereas IL-10 and IL-12 are anti-inflammatory cytokines, being a part of both antigen-specific adaptive and innate immune systems that inhibit proinflammatory response through limiting the damage to the host and maintaining tissue homeostasis (73, 74). As shown in Fig. 4A and 4B, NRG1 knockdown in LCs significantly (*P* > .05) increased IL-1β, IL-6, and IL-17α expression (see Fig. 4A and 4B). Since TNFα indirectly promotes inflammation by inducing cell death (68), in further analysis, NRG1 knockdown LCs were evaluated in the presence of exogenous TNFα. The treatment of TNFα enhanced expression levels of IL-1β, IL-6, and IL-17α both in scrambled and NRG1 knockdown LCs, whereas expression levels of IL-1β, IL-6, and IL-17α were significantly higher in endogenous NRG1 knockdown LCs. In contrast, the levels of IL-10 and IL-12 expressions were significantly decreased in LC with the knockdown of NRG1 (Fig. 4C and 4D). The treatment of TNFα significantly decreased the expression levels of IL-10 and IL-12 both in scrambled and NRG1 knockdown groups. The expression levels of IL-10 and IL-12 in NRG1 knockdown groups were much lower than in the scrambled groups.

Further analysis revealed that StAR protein expression decreased significantly with the treatment of TNFα both in scrambled and NRG1 knockdown groups (Fig. 4E and 4F). The expression levels of StAR in NRG1 knockdown groups in the presence of TNFα are much lower than in the scrambled groups. However, there were no statistically significant differences in StAR expressions in either the scrambled or NRG1 knockdown groups.

### Knockdown of Endogenous Neuregulin-1 With the Exogenous Treatment of Tumor Necrosis Factor α in Luteal Cells Inhibits the Phosphorylation of ErbB2/3, Phosphatidylinositide 3 Kinase –Protein Kinase B, and Extracellularly Regulated Kinase 1/2

**Figure 5.**
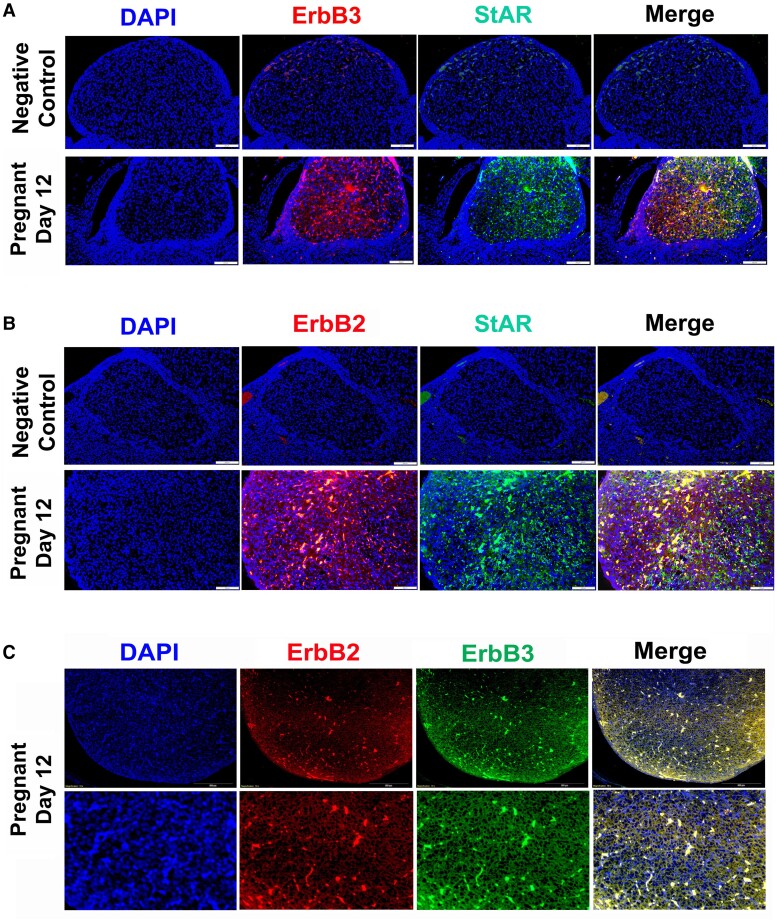
Ovaries were collected from pregnant rats on day 12 for immunohistochemistry (IHC) (n = 3 rats per group). A, Representative photomicrographs view of corpus luteum (CL) with immunolocalization of epidermal growth factor receptor 3 (ErbB3) and steroidogenic acute regulatory protein (StAR) with Alexa Fluor 594– (red) and Alexa Fluor 488– (green) labeled secondary antibodies, respectively. The nucleus was stained with DAPI (4′,6′-diamidino-2-phenylindole) (blue). B. Representative photomicrographs view of CL with immunolocalization of ErbB2 and StAR with Alexa Fluor 594– (red) and Alexa Fluor 488– (green) labeled secondary antibodies, respectively. The nucleus was stained with DAPI (blue). C, Representative photomicrographs view of CL with immunolocalization of ErbB3 and ErbB2 with Alexa Fluor 594– (red) and Alexa Fluor 488– (green) labeled secondary antibodies, respectively. The nucleus was stained with DAPI (blue).

**Figure 6.**
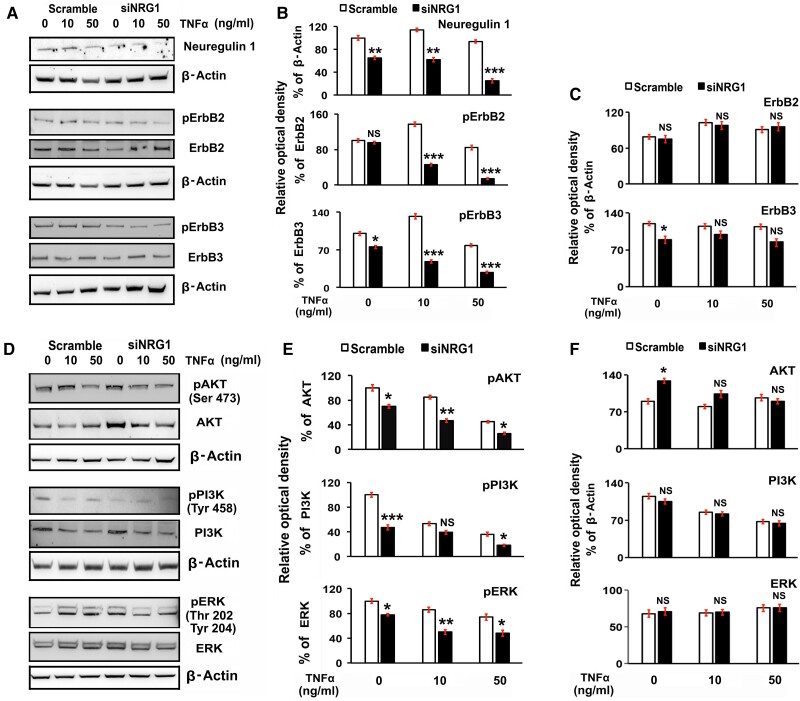
Effects of knockdown of endogenous neuregulin 1 (NRG1) on the exogenous treatment of tumor necrosis factor α (TNFα) on phospho(p)- and total ErbB2, ErbB3, phosphoinositide 3-kinase (PI3K), serine/threonine-protein kinase (AKT/protein kinase B), and extracellular signal-regulated kinase (ERK1/2) protein expressions in rat luteal cells (LCs). LCs were grown at 70% to 80% confluency and transiently transfected with small interfering-NRG1 (siNRG1) or scrambled RNA. After that, cells were maintained in serum-starved media and treated with TNFα at different concentrations (10 and 50 ng/mL) for 24 hours. A and D, Expression of phospho(p)- and total ErbB2, ErbB3 PI3K, AKT/PKB, and ERK1/2 protein expressions were determined by Western blot analysis. To each lane of the gel equal amounts of protein were applied. β-Actin was used as an internal control. Bar diagrams B to F represent the densitometric protein analyses in immunoblots (WBs). All bar graphs represent the mean ± SEM of results from 3 individual experiments (n = 3). Asterisks represent unpaired *t* test, *P less than or equal to .01; **P less than or equal to .001; ***P less than or equal to .0001; and NS, not significant.

Previous studies have established that NRG1 binding with ErbB3 heterodimerize with ErbB2 promotes autophosphorylation and transphosphorylation of tyrosine residue and activation of downstream signaling pathways (mainly PI3K/Akt and MAPK) to govern distinct cell-fate decisions (13, 42-48, 63-65, 75). Moreover, the present study showed an intense expression of NRG1 along with StAR in LCs in mid-cycle CL (see Fig. 1), which decreases significantly in regressive CL and inversely correlated with higher levels of TNFα expression prior to structural luteolysis (37). Therefore, to explore the NRG1 signaling in LCs, we first determined the expression pattern of ErbB2 and ErbB3 in LCs of mid-cycle CL. As shown in Fig. 5A and 5B, both ErbB2/3s were intensely colocalized in LCs along with StAR in mid-cycle CL. Further IHC analysis showed (Fig. 5C) that both ErbB2 and ErbB3 are intensely colocalized in mid-cycle CL. These IHC analyses suggest a possible role for ErbB2/3 in LCs of mid-cycle CL. Therefore, we explored whether the knockdown of endogenous NRG1 in LCs treated with exogenous TNFα affected the phosphorylation status of ErbB2 and ErbB3 and downstream signaling pathways, namely PI3K-AKT and ERK. As shown in Fig. 6A and 6B, the phosphorylation of the receptors ErbB2 and ErbB3 were significantly downregulated in the Nrg1 knockdown LCs, and phosphorylation was further downregulated when treated with TNFα compared with those of the control scramble groups. In further studies, we elucidate the effects on downstream signaling pathways of ErbB receptors under these experimental conditions. As shown in Fig. 6D and 6E, the expression of the phosphorylated PI3K/AKT and ERK1/2 was significantly downregulated in the Nrg1 knockdown LCs, and phosphorylation was further downregulated when treated with TNFα compared with those of the control scramble groups like ErbB2/3 phosphorylation. The depletion of NRG1 had a negative effect on the phosphorylation of ErbB2/3, PI3K, Akt, and Erk1/2 without changing the total ErbB2/3, PI3K/Akt, and Erk1/2 expression levels in LCs except ErbB3 and Akt at 48 hours post siNRG1 treatment (Fig. 6C and 6F). These results indicate that ErbB2 and ErbB3 are important receptors in LC functions.

## Discussion

The present study demonstrated a new role for NRG1 signaling as a protective effect against TNFα-dependent LC death. NRG1 expression in mid-CL is positively correlated with StAR, the functional marker of steroidogenesis, and E2 and P4 production, whereas it is negatively correlated with TNFα expression in regressive CL (6, 10, 37, 38, 76). The decrease of peripheral P4 levels characterizes functional luteolysis. In contrast, TNFα is a potential luteolytic cytokine that increases during functional luteal regression and postpartum CL and inhibits the action of luteotropic factors. These results agree with previously published results (7, 19, 37, 38, 40, 76-78). Thus, our present results suggest a strong functional role for NRG1 in mid-CL. Therefore, to understand the physiological role of NRG1 in LCs, we knocked down the endogenous NRG1 in LCs. The exogenous TNFα is used as a luteolytic agent for LC death in vitro. The knockdown of endogenous NRG1 promoted exogenous TNFα-induced activation of caspase 3/7 activity and proapoptotic factor Bak expression with LC death. TNFα coordinates tissue homeostasis by regulating cell survival and death by activating downstream effector caspases-3/7 (67, 78-81). Irrespective of the specific death-initiating stimulus, caspase-3 and -7 are activated universally during apoptosis (80). Under inflammatory conditions, the activation of caspase-7 is dependent on the inflammasome (caspase-1 complex) (80-83). On the other hand, caspase-3 activation is dependent on the caspase-8 and -9 protein complexes during apoptosis (81-83). Our present results suggest that the knockdown of endogenous NRG1 could not preserve the viability of LCs by tilting the balance in favor of apoptosis. The proapoptotic protein Bak counteracts the apoptosis-inhibiting effects of Bcl2 and Bclxl. A wide variety of cell-death stimuli, including excessive cytokines, causes an imbalance of the Bak vs Bcl2 and Bclxl ratios and tilts the scales toward cell death (43, 69, 83-85). Published studies demonstrated that the increase in caspase activity coincides with the decrease in luteal steroidogenic enzyme activities and steroidogenesis (38, 86). The endogenous NRG1 knockdown in GCs promoted early events of caspase-dependent cell death (38, 42, 43, 86). Thus, the present results suggest that endogenous NRG1 counterbalances the TNFα-induced LC death and is a prosurvival factor in LCs.

In additional studies, our results indicated that endogenous NRG1 might act as an essential regulator of IL expression in LCs similar to the GCs (42). This supposition is supported by numerous studies in which NRG1 signaling was demonstrated to regulate proinflammatory cytokines and chemokine in various cell types and distinct disease states (42, 43, 45, 46, 48). In our study, cytokine expression, especially IL-1β, IL-6, and IL-17α, is significantly augmented in NRG1 knockdown LCs as they play an important role in the CL regression phase (2-4). The uncontrolled expression of cytokines and chemokines modulates the immunoregulatory factors that can promote the inflammatory response (12, 14, 87, 88). The uncontrolled or abundant intraovarian IL expression adversely affects ovarian reserve with pathophysiological aging (88-91), including hormone production, ovulation, fertility, luteal phase insufficiency, and pregnancy (5, 9, 19, 38, 77, 92). Moreover, one cause of decreased luteolysis during the postpartum period with a prolonged anovulatory status is a lower concentration of inflammatory cytokines (5, 9, 19, 38, 77, 92). Other studies have demonstrated that exogenous NRG1 administration attenuates oxidative stress and inflammation by inhibiting the production of reactive oxygen species IL-1β and NLRP3 inflammasome (93), and supports cardiac repair, sepsis, traumatic spinal cord injury (50-57, 93-96). NRG1 levels are elevated in plasma and brain tissue of transgenic sickle cell disease and ischemic mice and prevent the expression of inflammatory cytokines, chemokines, and adhesion molecules (50-57, 93-96). These results suggest that NRG1 may have emerged as a critical molecular regulator of ILs to balance inflammation and prevent uncontrolled CL maturation or luteolysis (42, 52, 53, 57, 62-65, 75, 97).

Our further studies suggest that the anti-inflammatory and prosurvival effects of NRG1 in LCs are through ErbB2/3 by activating either the PI3K-AKT or ERK1/2 signaling pathway (42, 43, 63-65, 75). The colocalization of ErbB2/3 in the CL suggests that NRG1 may act as a ligand for ErbB2/3 in the CL (63-65, 75). The ErbB2-ErbB3 heterodimers represent the most potent signaling complexes enhanced by their binding to NRG1 and support various cellular functions, including gonadotropins-dependent NRG1-ErbB2/3 signaling in follicular maturation and ovulation (13, 42-48, 64, 69). The inverse correlation between phosphorylated ErbB2/3 and the activation of caspase 3/7 and proapoptotic factor Bak expression suggest that possibly knockdown of NRG1 affected the ErbB2/3 dependent phosphorylation of PI3K, AKT, and ERK1/2 (13, 42-48, 65, 98, 99). As a result, the knockdown of endogenous NRG1 was unable to preserve the viability of LCs by tilting the balance in favor of apoptosis due to inhibition of the PI3K/AKT/ERK1/2-signaling pathways through ErbB2/3 as in other cell types, including our previous studies (13, 42-48, 65, 98, 99). ErbB3 loss in the mammary epithelium of mice impaired Akt and MAPK signaling and reduced luminal cell survival and proliferation with increased expression of multiple cytokines (63). Moreover, PI3K/AKT is essential in inactivating Bak and inflammatory responses (13, 42-48, 65, 98, 99). Together, these results suggest that NRG1-ErbB2/3 signaling in LCs provides an extra layer in orchestrating the microenvironment for CL physiological competency during pregnancy.

In conclusion, the present findings provide new information on the local expression and action of NRG1 in LCs as an immunomodulatory and prosurvival factor that may be involved in affecting luteinization in mid-CL by preventing proinflammatory cytokines associated functional regression of CL, which ultimately supports P4 production and fine-tunes CL differentiation. However, several other mechanisms may regulate cytokine expression and action in LCs and the CL (100).

## Data Availability

The authors confirm that the data supporting the findings of this study are available within the article, except small interfering NRG1 (siNRG1, sequence will be available upon request). Therefore, any other declaration is “not applicable.”

## Prior Presentation

Part of this work was presented at the ENDO 2021 (Virtual Conference), Endocrine Society, Washington DC, USA, March 22-March 23, 2021; Research Centers in Minority Institutions (RCMI) Consortium National Conference, Virtual Conference, USA (March 24-26, 2021, and March 14-18, 2022); the 54th Annual Meeting of the Society for the Study of Reproduction, St Louis, MO, USA (December 14-18, 2021); and the 55th Annual Meeting of the Society for the Study of Reproduction, Spokane, Washington, USA (July 26-29, 2022).

## Abbreviations

Akt: Ak strain transforming or protein kinase B
Bcl: B-cell lymphoma
BW: body weight
CL: corpus luteum
DAPI: 4′,6-diamidino-2-phenylindole
E2: estrogen
EGF: epidermal growth factor
ERK: extracellularly regulated kinase
FBS: fetal bovine serum
GCs: granulosa cells
H&E: hematoxylin-eosin
IHC: immunohistochemistry
IL: interleukin
LCs: luteal cells
LH: luteinizing hormone
MAPK: mitogen-activated protein kinase
mRNA: messenger RNA
NIH: National Institutes of Health
NRG1: neuregulin 1
P4: progesterone
PCR: polymerase chain reaction
PI3K: phosphatidylinositide 3 kinase
siNRG1: small interfering NRG1
siRNA: small interfering RNA
StAR: steroidogenic acute regulatory protein
TNFα: tumor necrosis factor α
TNFR1: TNF receptor 1
vWF: von Willebrand factor
WB: Western blot
